# Cavernous hemangioma in the thymus: a case report

**DOI:** 10.1186/s40792-016-0137-6

**Published:** 2016-02-10

**Authors:** Naoko Ose, Yuko Kobori, Yukiyasu Takeuchi, Yoshiyuki Susaki, Seiji Taniguchi, Hajime Maeda

**Affiliations:** Department of General Thoracic Surgery, National Hospital Organization Toneyama Hospital, 5-1-1 Toneyama, Toyonaka-shi, Osaka, 560-8552 Japan

**Keywords:** Cavernous hemangioma, Thymus, Surgery, Partial resection, Image analysis system

## Abstract

Cavernous hemangioma is not a neoplasm, but rather a congenital venous malformation with the potential to develop in all parts of the body, though it is very rarely seen in the thymus. We report a case of cavernous hemangioma in the thymus partially resected. A 71-year-old woman presented with pericardial discomfort, and chest computed tomography (CT) showed a left lateral mediastinal mass which was 2.0 × 1.2 × 1.8 cm in size, with border regularity and without calcification. Its interior was partially enhanced. Three-dimensional chest computed tomography image showed a tortuous vessel connecting to the tumor. Surgical resection was performed for the purpose of providing a definitive diagnosis and treatment because a mediastinal tumor such as thymoma or teratoma was suspected. Partial resection of the thymus including the mass was done by utilizing a three-port, left-sided video-assisted thoracic surgery (VATS) approach with hoisting of the third rib with the patient in a spinal position. A wine-colored mass bulging from the surface of the left lobe of the thymus was identified along with the communicating vessel which could only be cut with an energy device. It is considered that thymic partial resection using VATS is a better option for small and non-infiltrative lesions.

## Background

Cavernous hemangioma (CH) is not a neoplasm, but rather a congenital venous malformation with the potential to develop in all parts of the body, though it is very rarely seen in the thymus. Here, we report a case of cavernous hemangioma in the thymus that was partially resected using a video-assisted thoracic surgery (VATS) procedure.

## Case presentation

**Fig. 1:**
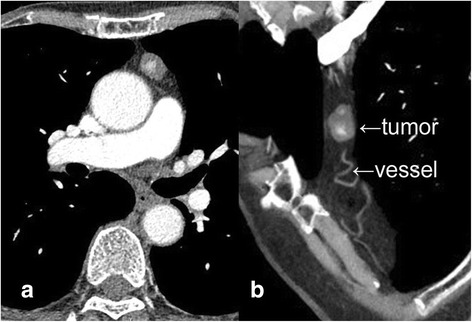
Enhanced chest computed tomography (CT) scan showing **a** anterior mediastinal tumor in the left lobe of thymus, 2.0 × 1.2 × 1.8 cm in size, and **b** vein running in the thymus in communication with the tumor (*arrow*)

A 71-year-old woman presented with pericardial discomfort while coughing, and chest computed tomography (CT) showed a left lateral mediastinal mass. Although the mass remained unchanged in CT findings 3 months later, surgical resection was recommended because a mediastinal tumor such as thymoma or teratoma was suspected. The anterior mediastinal mass was 2.0 × 1.2 × 1.8 cm in size, with border regularity and without calcification. Its interior was partially enhanced (Fig. 1a), and a tortuous vessel was revealed in the thymus in enhanced CT imaging (Fig. 1b).

**Fig. 2:**
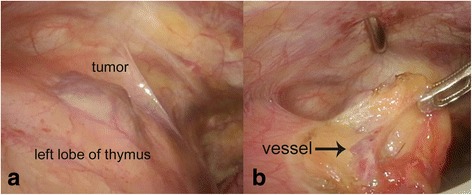
Intraoperative findings. **a** Dark red tumor bulging from the surface of the thymus. **b** The connecting vessel (*arrow*) was cut using an energy device

Surgical resection was performed for the purpose of providing a definitive diagnosis and treatment. With the patient in a spinal position, partial resection of the thymus including the mass was done by utilizing a three-port, left-sided VATS approach with hoisting of the third rib. A wine-colored mass bulging from the surface of the left lobe of the thymus was identified (Fig. 2a) along with the communicating vessel (Fig. 2b), which could only be cut with an energy device. The operative time was 138 min and there was only minor blood loss. The patient was discharged on postoperative day 7 without complications.

**Fig. 3:**
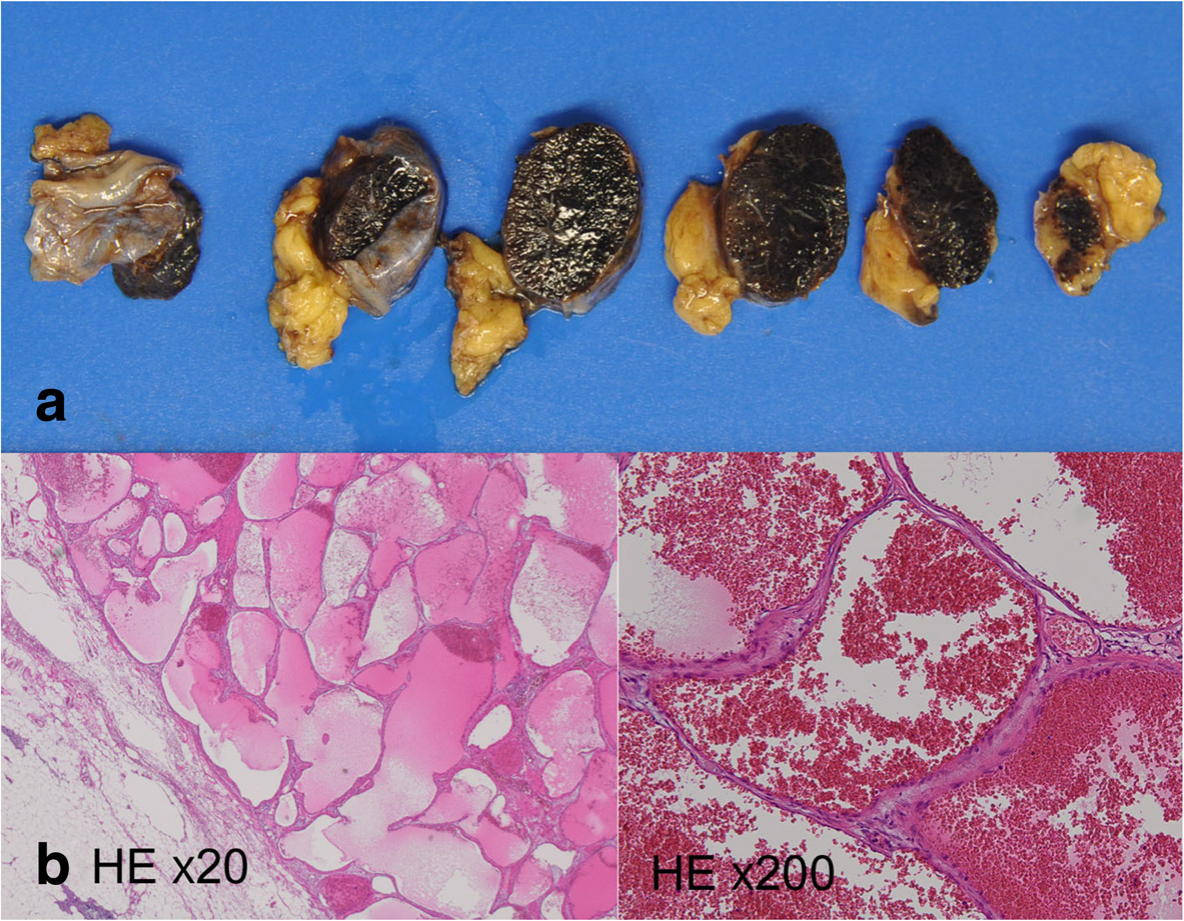
Histopathological findings of the tumor used for diagnosis of cavernous hemangioma. **a** There was no invasion of the adjacent thymus. **b** Hematoxylin and eosin (HE) staining showed a blood vessel ingredient, indicating expansion, and filling with a blood constituent

The size of the excised mass was 2.3 × 1.3 × 1.8 cm, and there was no infiltration of the adjacent thymus (Fig. 3a). A histopathological diagnosis of CH in the thymus was made based on the marked dilated vascular channels full of blood covered with endothelial cells and without atypia (Fig. 3b), and the vessel was included in the mass.

### Discussion

CH is not a vascular tumor, but rather a congenital vascular anomaly classified as a venous malformation in the low-flow lesion category by the International Society for the Study of Vascular Anomalies (ISSVA) [1, 2]. This abnormality can occur throughout the whole body, though size and site of origin vary. Some reports have noted its occurrence in patients with a venous aneurysm [3, 4]. Such a vascular anomaly is considered to be the result of local abnormal morphogenesis of normal vascular endothelial cells occurring before birth [2], which then increases in size and exists for the lifetime of the individual without atrophy. Approximately half of the patients with CH are diagnosed at younger than 20 years old. Histopathological findings generally show cavernous or cystic venous channels with a flat single layer of endothelial cells without atypia or mitosis. The choice of treatment is dependent on the related organ and can include embolization, sclerotherapy, and surgical resection. In cases of resection, the lesion should be completely removed because residual tissue has the potential for recurrence with abnormal vessel dilation or regeneration.

CH occurs with greater frequency in the liver, accounting for approximately 30 % of known cases, as well as in the brain and skin, whereas there are several reports of mediastinal cases in Japan [5]. Since it was previously thought to be a tumor, a mediastinal CH is considered to be rare, accounting for 0.5 % or less of all mediastinal tumors [6]. Furthermore, there are only seven reports of cases with a proven thymic origin [3, 4, 7–9].

When occurring in the thymus, a CH may require resection for diagnosis and treatment, as it is otherwise difficult to distinguish from another type of mediastinal tumor such as a thymoma. Even though it does not generally invade the neighboring tissue, complete resection is desirable, as previously described. Cohen et al. reported that subtotal excision should be chosen if complete resection carries serious risk because there was no rapid growth noted in a case of incomplete resection and re-resection can be considered at the time of recurrence [6]. Extirpation by means of a median sternotomy or thoracotomy will be needed in cases with a giant mass [9] or innominate vein aneurism [3, 4]. On the other hand, thymic partial resection using VATS is a better option for small and non-infiltrative lesions, such as seen in the present case.

**Fig. 4:**
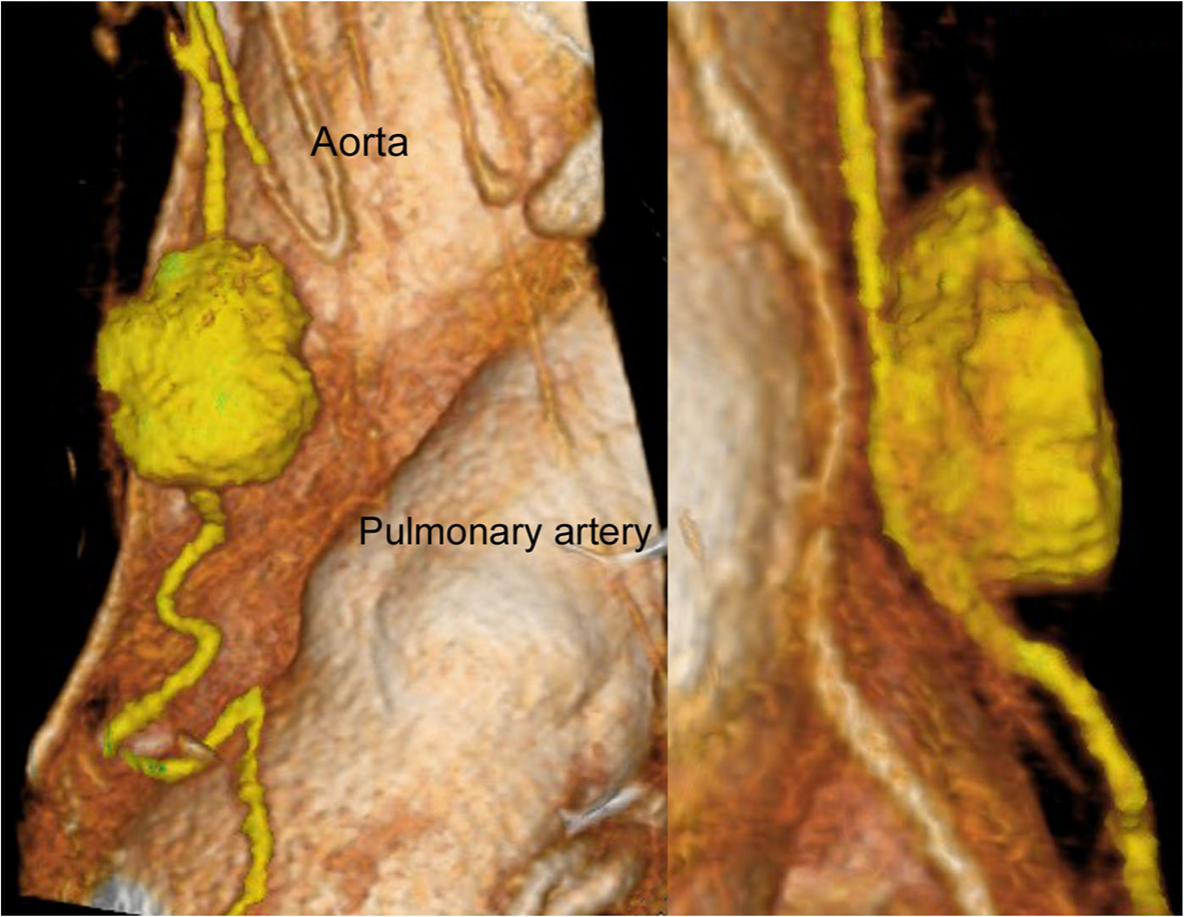
Three-dimensional chest computed tomography (3D-CT) image showing a tortuous vessel connecting to the tumor

For accurate diagnosis of CH, magnetic resonance (MR) imaging or ultrasound sonogram are often used to examine the brain, liver, and other organs. The typical imaging manifestation of CH is popcorn-like lesion with a peripheral hypointense rim so-called hemosiderin rim on T2-weighted MR images. As for mediastinal or lung vascular anomalies, enhanced CT may be very effective, as it can reveal a phlebolith in some cases of venous malformation. No phlebolith was seen in our patient, while the center of the mass was enhanced and a communicating vessel running through the thymus was detected. In addition, findings obtained with a three-dimensional image analysis system (SYNAPSE VINCENT ver. 4.0, Fujifilm Medical Inc., Japan) showed the positional relationship of the tortuous vessel and the mass on the side wall (Fig. 4). We consider that such an image analysis system is helpful for diagnosis of vessel malformation, as seen in the present case.

The mass was resected completely but we intend to follow the present patient to note vessel changes.

## Conclusions

We experienced a rare case of CH in the thymus. Our experience indicated that enhanced CT and a three-dimensional image analysis system would be helpful for diagnosis of vessel malformation and thymic partial resection using VATS would be a better option for small and non-infiltrative lesions.

### Consent

Written informed consent was obtained from the patient for the publication of this report and any accompanying images.

## Abbreviations

CH: cavernous hemangioma
CT: chest computed tomography
VATS: video-assisted thoracic surgery
